# Cannabinoid Hyperemesis Syndrome: A Rising Complication

**DOI:** 10.7759/cureus.78958

**Published:** 2025-02-13

**Authors:** Saar Peles, Roy Khalife, Anthony Magliocco

**Affiliations:** 1 Gastrointestinal Oncology, University of Central Florida College of Medicine, Orlando, USA; 2 Genetics, Protean Biodiagnostics, Orlando, USA; 3 Pathology, University of Central Florida College of Medicine, Orlando, USA

**Keywords:** cannabinoid hyperemesis syndrome (chs), cannabis use, cyp system, emergency medical service, substance-induced disorders

## Abstract

Cannabis, commonly known as marijuana, is a psychoactive plant that has been used for both medicinal and recreational purposes for centuries. It contains over 100 biologically active compounds known as cannabinoids, the most notable of which are tetrahydrocannabinol (THC) and cannabidiol (CBD). THC is responsible for the euphoric and hallucinogenic effects associated with cannabis use, while CBD is often utilized for its potential therapeutic benefits, such as pain relief and anti-inflammatory properties. Despite its widespread reputation for alleviating nausea and stimulating appetite, chronic cannabis use has been linked to a paradoxical condition known as cannabinoid hyperemesis syndrome (CHS). CHS is a disorder that paradoxically causes abdominal pain, nausea, and uncontrollable vomiting in long-term cannabis users rather than alleviating pain and reducing nausea. Misdiagnosis of this condition is extremely common, and it is often confused with cyclic vomiting syndrome (CVS). The underlying pathogenesis of CHS is not completely understood, though several mechanisms have been proposed. Although considered rare, there has been a steady increase in CHS diagnoses in the Emergency Department (ED). This article summarizes the symptoms, pathogenesis, treatments for CHS, and differential diagnoses to further increase our understanding of this condition.

## Introduction and background

Cannabis, commonly referred to as marijuana, is a plant from the Cannabis sativa species that has been cultivated and consumed for medicinal, industrial, and recreational purposes for thousands of years. The plant’s psychoactive and medicinal effects are attributed to its cannabinoids, chemical compounds that interact with the body's endocannabinoid system. Among these cannabinoids, tetrahydrocannabinol (THC) is the most well-known for its ability to induce euphoria, alter perception, and stimulate appetite. Cannabidiol (CBD), another major cannabinoid, has gained attention for its potential therapeutic properties, including anti-inflammatory, anxiolytic, and neuroprotective effects [1].

Until the past decade, marijuana, specifically THC, had been largely illegal to possess and use, and its legalization has prompted new medical insights into its effects, both positive and negative. The use of marijuana has surged significantly in recent years, fueled by legalization efforts and increasing societal acceptance. As of December 2024, all but four states have full or partial legal marijuana laws or decriminalization laws in effect [2]. 2.6 million Americans become new users each year; the majority of this group is under the age of 19 [3]. Marijuana vape pens have also made it more convenient for young people, with a significant increase of 10th and 12th graders using this method in 2020 compared to 2019, according to the National Institute on Drug Abuse [4]. With around 15% of Americans currently using marijuana and the industry rapidly booming, it is important to note any health concerns that may arise through cannabis usage.

While cannabis has long been associated with its anti-nausea and pain-relieving properties [1], it also presents paradoxical effects in some long-term users. One such adverse effect is cannabinoid hyperemesis syndrome (CHS), a condition characterized by recurrent nausea, vomiting, and abdominal pain. First documented in 2004 by Allen et al. [5], CHS was initially thought to be a rare disorder. However, as cannabis use has increased, particularly in regions with legalized markets, CHS has become an emerging public health concern. The syndrome presents a unique contradiction: while cannabis is widely used to combat nausea, chronic use can, in some cases, lead to severe cyclic vomiting episodes that are only resolved by cannabis cessation. This comprehensive review explores the epidemiology, pathophysiology, diagnosis, treatment, and public health implications of CHS, aiming to bridge knowledge gaps and provide directions for future research.

## Review

Literature selection criteria

For this narrative review, relevant studies on CHS and its associated factors were identified through a comprehensive search of PubMed, Google Scholar, and other academic databases up to January 2025. Studies were included based on the following criteria.

Study Type

Studies eligible for inclusion included peer-reviewed articles, randomized controlled trials, observational studies, case reports, clinical trials, and systematic reviews focusing on CHS or comparing it with similar disorders like cyclic vomiting syndrome (CVS). Studies exploring novel treatments, diagnostic criteria, or the pathophysiology of CHS were also prioritized.

Only peer-reviewed articles, case reports, clinical trials, and review articles focusing on CHS or its comparison with similar disorders (e.g., CVS) were considered. Both primary research and secondary literature, such as systematic reviews and meta-analyses, were included to provide a broad perspective on the condition.

Language

Articles published in English were included to maintain consistency and ensure accessibility of the studies. Non-English publications were excluded unless an English abstract was available.

Population

The studies included focused on individuals diagnosed with CHS, as well as those with comparable conditions such as CVS, and chronic cannabis users exhibiting symptoms similar to CHS. A key focus was on studies that included detailed demographic information such as age, gender, and cannabis usage patterns, as well as data regarding co-morbidities, substance use history, and prior treatments.

Studies focused on individuals diagnosed with CHS, as well as those with comparable conditions like CVS, were selected. Emphasis was placed on studies that reported on demographic information, symptom patterns, diagnostic criteria, and treatments.

Exclusion Criteria

Studies that were purely theoretical without clinical data or those that focused exclusively on animal models were excluded, as the review aimed to capture human clinical insights into CHS. Additionally, studies that did not discuss cannabis use in relation to CHS symptoms were not considered.

After applying these criteria, a selection of relevant studies was included in this narrative review. These studies were analyzed and synthesized to provide an overview of CHS, its clinical features, and the evolving understanding of its relationship to chronic cannabis use.

Epidemiology

Research indicates the prevalence of CHS has risen in tandem with increasing cannabis use, particularly among chronic users. For example, a study in an urban New York City Emergency Department consisting of 2127 patients showed that 32.9% of patients who smoked marijuana 20 days or more per month met the criteria for CHS [6]. While this study acknowledges limitations due to self-reported usage and symptoms, if extracted to the general population, between 2.13-3.38 million suffer from CHS. In Colorado, a 2015 study found that CHS-related visits doubled following legalization in 2009 [7]. Similarly, a study following trends for CHS-related emergency department visits in Nevada before and after legalization found an increase from 1.07 per 100,000 visits to 2.22 per 100,000 [8]. Another study by Angulo in 2024 also found that between 2017 and 2021, CHS-related emergency department visits doubled in the United States and Canada most commonly among males between 16 and 34 of age [9]. 

The general consensus among studies from states that have legalized marijuana is that cases of CHS presenting at hospitals have doubled post-legalization. However, the true prevalence is likely underestimated due to misdiagnosis and limited awareness among healthcare providers. While it is possible that legalization has made patients more willing to seek emergency care, the burden of managing these cases ultimately falls on healthcare providers. Moreover, cannabis legalization has introduced higher-potency products to the market, which may be a significant factor contributing to the rising incidence of CHS.

Diagnosis: Current Challenges and Emerging Solutions

CHS is commonly divided into three distinct phases: the prodromal, hyperemetic, and recovery phases. The prodromal phase is characterized by morning nausea and abdominal discomfort, and it can persist anywhere from four months to five years. The hyperemetic phase is marked by severe, intractable nausea and vomiting, accompanied by abdominal pain, which generally does not respond to antiemetic treatments. During this phase, patients often develop a reluctance to consume solid foods, as ingestion typically triggers vomiting. Finally, the recovery phase occurs when patients return to their baseline health status, regardless of whether they resume cannabis use. CHS is primarily induced by chronic cannabis consumption, with no identifiable organic cause, and the condition is typically managed through the cessation of cannabis use.

According to Sorensen et al. [10], the key diagnostic characteristics of patients with CHS include severe nausea and vomiting (100%), recurring cyclic vomiting over months (100%), below 50 years of age at diagnosis (100%), frequent cannabis use, at least weekly (97.4%), symptom resolution after cannabis cessation (96.8%), compulsive hot showers or baths that provide symptom relief (92.3%), abdominal pain (85.1%), history of daily cannabis use (76.6%), history of regular cannabis use for over a year (74.8%), male predominance (72.9%).

CHS typically affects individuals younger than 50 years, with a median age of 28 years. Notably, the median age for cannabis initiation in these patients is 16, suggesting that early cannabis use may play a role in CHS development.

Despite these established characteristics, there is no definitive guideline on how many criteria are required for diagnosis. CHS episodes generally last a few days but can persist for up to seven to 10 days. Patients may report that hot water alleviates symptoms, causing some to spend several hours in the shower [11]. 

Most CHS patients present to the emergency department multiple times, often undergoing various diagnostic tests, including lab work and advanced imaging, which typically yield negative results. Common clinical findings include signs of dehydration, electrolyte imbalances, and ketonuria following episodes of severe nausea and vomiting.

Challenges in Differential Diagnosis

The difficulty in diagnosing CHS arises because its symptoms overlap with other gastrointestinal and metabolic disorders, such as gastroparesis, cyclic vomiting syndrome, or peptic ulcer disease. Overlapping symptoms such as abdominal pain and cyclic vomiting are especially difficult to pinpoint to a specific illness.

This often leads to misdiagnosis and delayed treatment that could otherwise alleviate symptoms. The absence of specific biomarkers for CHS means that physicians must rely primarily on clinical history, which can be challenging when the patient does not openly disclose cannabis use or when cannabis use is intermittent.

Clinical Diagnosis Versus Laboratory and Imaging Findings

Although laboratory examinations and advanced imaging studies (e.g., CT scans, ultrasounds) are often negative, they play a crucial role in ruling out other conditions. The lack of significant diagnostic findings in CHS patients underscores the importance of a thorough patient history and clinical suspicion. Despite negative workups, physicians must consider CHS when standard treatments fail and patients continue to present with persistent symptoms related to cannabis use.

Emerging Diagnostic Tools

Recent studies have explored the potential use of biomarkers in diagnosing CHS. For example, elevated serum cannabinoid levels or certain patterns of urinary metabolites could help confirm chronic cannabis use, though further research is needed to validate these approaches. Additionally, genetic research is ongoing to identify potential genetic markers that may predispose individuals to CHS, which could contribute to a more precise diagnosis.

Role of Patient History in Diagnosis

Patient history remains the most reliable tool in diagnosing CHS. As the condition is tied closely to long-term cannabis use, comprehensive patient interviews should include inquiries about frequency, potency, and duration of cannabis use, as well as the onset and progression of symptoms. While the literature on these exact criteria is sparse, consistently heavy use for long periods of time, makes CHS as a result of marijuana use much more likely. Patients may not initially associate their symptoms with cannabis, so creating an open and non-judgmental environment is crucial to obtaining accurate information.

Psychosocial Factors

Psychosocial factors may also influence the diagnosis and reporting of CHS. The stigma surrounding cannabis use, especially in populations who consume cannabis regularly, can deter patients from disclosing their usage to healthcare providers. Therefore, cultivating a more accepting healthcare environment for patients using cannabis could improve the accuracy of self-reported data and help in the earlier identification of CHS.

The Need for Standardized Guidelines

The absence of standardized diagnostic criteria for CHS is a significant challenge in clinical practice. Standardized guidelines would help healthcare providers identify CHS more efficiently and reduce the likelihood of misdiagnosis. The development of such guidelines would not only benefit clinicians but could also improve patient outcomes by ensuring timely and accurate treatment.

While clinical features such as chronic cannabis use, intractable vomiting, and relief with hot baths are commonly reported, these are not pathognomonic. The development of a validated CHS diagnostic tool, potentially incorporating biomarkers like cannabinoid metabolites or genetic polymorphisms, could revolutionize early detection and management.

Cannabinoid hyperemesis syndrome versus cyclic vomiting syndrome

CHS is frequently confused with CVS due to overlapping clinical features. Both conditions involve recurrent episodes of acute nausea and vomiting, as well as compulsive hot showers, but the key distinction lies in cannabis use, which is a hallmark of CHS but not associated with CVS [12]. Researchers continue to debate whether CHS and CVS represent distinct disorders or if CHS is a subset of CVS. For CHS and CVS to be considered separate entities, patients must exhibit different clinical characteristics, affect different populations, or respond to treatments in divergent ways.

Since the Department of Justice issued a memo that it would not prosecute marijuana users and sellers who complied with state law in 2009, hospital discharges for compulsive vomiting have increased by 8% annually [13]. This rise may, in part, be due to recognition bias, as emergency department (ED) physicians fail to recognize cyclic vomiting in more than 80% of cases [14]. Both conditions predominantly affect younger individuals, with CHS showing a higher prevalence in males, which aligns with the higher rates of cannabis use among men. While CVS tends to be more common in women according to some studies, gender biases in both conditions remain inconclusive [15].

Abdominal pain is a key feature of CHS, although it is also present in a significant proportion of CVS cases [16]. Some researchers have proposed compulsive bathing or showering in hot water be considered a mandatory diagnostic criterion for CHS, as this behavior appears to alleviate symptoms. However, 10% of CHS patients do not report this behavior, despite it being central to the diagnosis [11,17]. A comparison study further questions the specificity of this symptom, revealing that 48% of CVS patients who do not use cannabis report relief from hot baths, compared to 72% of cannabis users [17]. While hot baths are strongly associated with cannabis use in most studies, this association is not pathognomonic [18]. 

Both CHS and CVS are frequently misdiagnosed and likely more common than previously acknowledged. Table 1 highlights the characteristics of each condition in an attempt to differentiate them. Beyond cannabis use, genetic predisposition plays a crucial role in CHS development. Emerging research highlights the potential involvement of mutations in the cytochrome P450 (CYP) enzymes, which affect cannabinoid metabolism. By contrast, CVS is often associated with stress or migraine comorbidities, underscoring the need for tailored diagnostic criteria. Genetic studies may possibly reveal that long-term cannabis use triggers symptoms in patients genetically predisposed to developing CVS [19].

**Table 1:** Characteristics of Cannabinoid Hyperemesis Syndrome (CHS) and Cyclic Vomiting Syndrome (CVS) Data sourced from reference [12]

	CHS	CVS
Demographics	Majority Caucasian, 7:3 male:female ratio	Majority Caucasian, 3:2 male:female ratio
Pre-req for Suspicion/Diagnosis	Chronic cannabis user	Migraine and/or psychiatric comorbidities
Triggers	Cannabis usage, stress	Stress, pleasant excitement, infections, menstrual periods
Symptoms	Delayed gastric emptying, abdominal pains, nausea, vomiting	Quick gastric emptying, abdominal pains, nausea, vomiting
Treatment (prodromal phase)	Cannabis cessation	Medication to abort episode (e.g. metoclopramide and ondansetron)
Follow-up Treatment	Psychiatric treatment for cannabis cessation	Avoiding trigger-factors and application of preventative medication

Pathophysiology and molecular insights

A key question in understanding CHS is the underlying cause of its development. Pergolizzi et al. [20] provide an in-depth exploration of the pathogenesis of CHS. Cannabis contains over 100 different cannabinoids, with delta-9-THC and CBD being the primary compounds. These cannabinoids bind to cannabinoid receptors type 1 (CB1) and type 2 (CB2), which are distributed throughout the body. Specifically, CB1 receptors are primarily located in the brain, while CB2 receptors are found outside the central nervous system (CNS), in areas like the spleen, thymus, and other immune cell populations [21].

THC, the principal exogenous cannabinoid in cannabis, is metabolized in the liver through oxidative and hydroxylation reactions by the CYP2C enzyme subfamily [22]. THC is known for its antiemetic properties when it binds to both CB1 and CB2 receptors. The half-life of THC ranges from 20 to 30 hours, though this can vary depending on the cannabis product [23]. THC is excreted in feces (60-85%) and urine (20-35%) as acid metabolites. Due to its lipophilic nature, THC accumulates in adipose tissue over time [24]. This storage creates a "reintoxication" effect, where stored THC can be released under conditions such as stress or prolonged fasting [24]. Chronic cannabis users tend to have significant THC reserves in their fat tissue, which can be mobilized during stressful situations.

Other cannabinoids, such as CBD and cannabigerol (CBG), may also contribute to the development of CHS. At low doses, CBD is known to be antiemetic, but at higher doses, it can become proemetic. CBG has the potential to reverse the antiemetic effects of CBD, suggesting that CHS could result from the interplay between high levels of CBD and its reversal by CBG [25,26].

**Figure 1:**
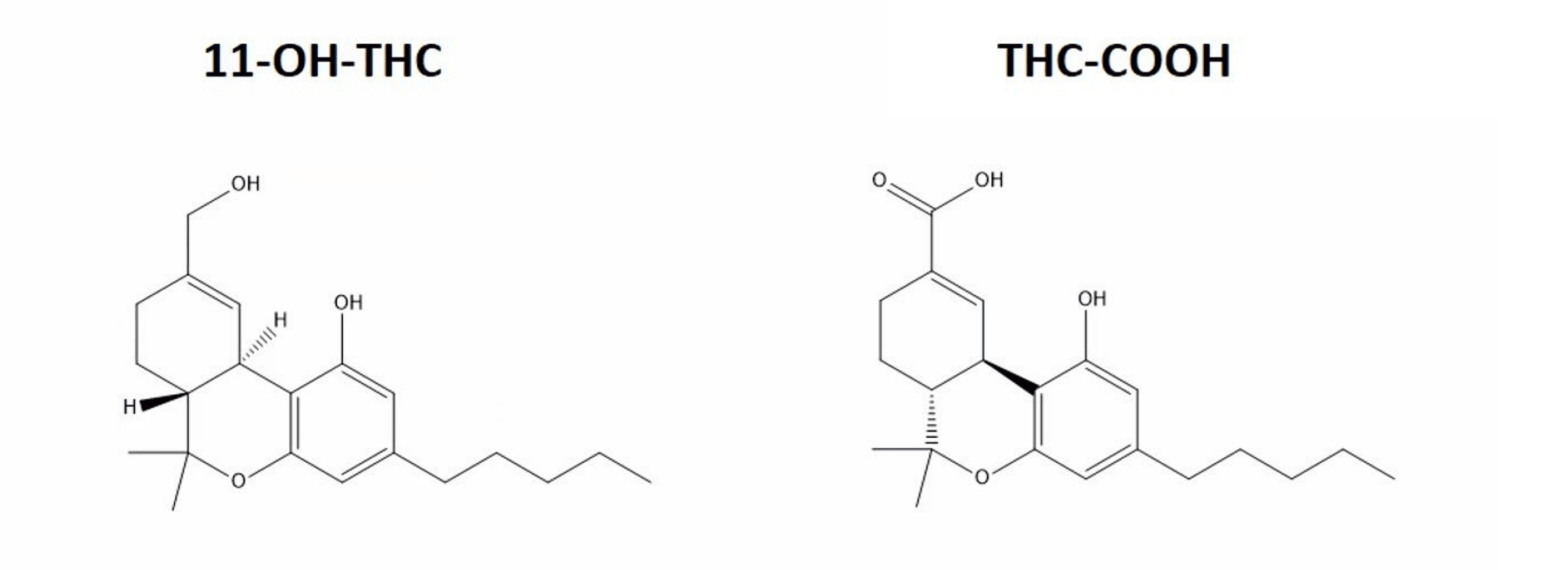
The main two metabolites of tetrahydrocannabinol (THC) are shown in this figure. If a drug-transforming enzyme is unable to properly metabolize these, it can lead to accumulation and possibly toxicity in the body. This figure was made on ChemDraw by Roy Khalife. 11-OH-THC: 11-hydroxy-delta9-tetrahydrocannabinol, THC-COOH: 11-nor-9-carboxy-THC-delta9-tetrahydrocannabinol

The susceptibility to CHS may also be influenced by cytochrome CYP450 metabolism and genetic polymorphisms. Variations in genes encoding enzymes like CYP2C9, CYP2C19, and CYP3A4 can lead to an excess accumulation of cannabinoid metabolites, potentially triggering vomiting. THC is primarily metabolized to two major metabolites, 11-hydroxy-delta9-tetrahydrocannabinol (11-OH-THC) and 11-nor-9-carboxy-THC-delta9-tetrahydrocannabinol (THC-COOH), illustrated in Figure 1, along with over 100 minor metabolites [27]. 11-OH-THC is psychoactive and shares similar potency to THC, whereas THC-COOH is non-psychoactive and has anti-inflammatory effects [27-29].

**Figure 2:**
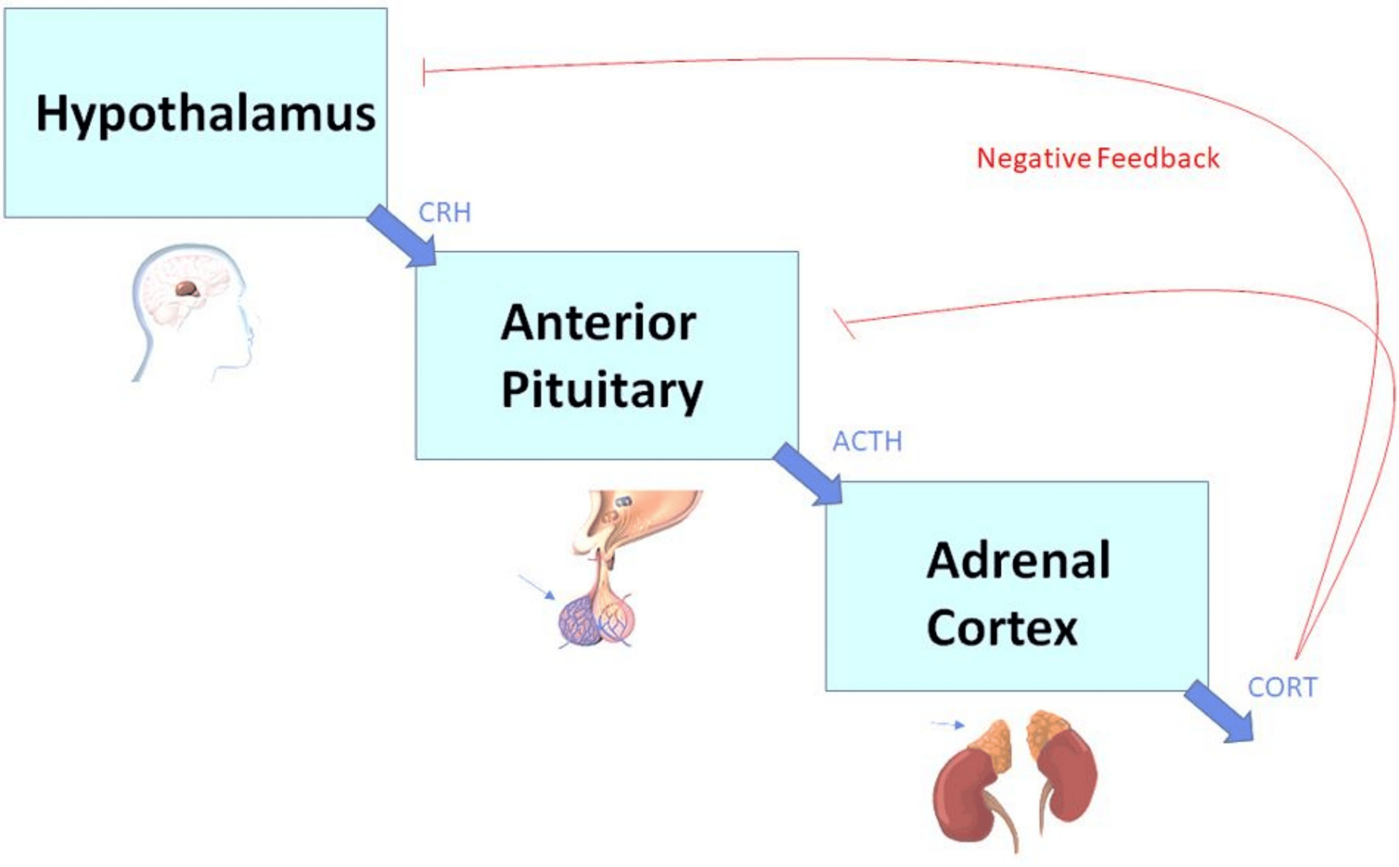
Illustrated above is the simplified flow of the hypothalamic-pituitary-adrenal (HPA) axis. Typically, regular amounts of tetrahydrocannabinol (THC) and cannabidiol (CBD) reduces the body’s response to stress, therefore lowering the amount of cortisol produced. However, excessive THC can disrupt homeostasis, raising cortisol levels to the point of harm. Image Credits: Roy Khalife CRH: corticotropin releasing hormone, ACTH: adrenocorticotropic hormone, CORT: cortisol

If the endocannabinoid system gets disrupted by excessive use of cannabinoids, the stimulation of the hypothalamic-pituitary-adrenal (HPA) axis and the sympathetic nervous system may occur. Figure 2 portrays the flow of hormones released when stress occurs. Stress is regulated and controlled partially by the endocannabinoid system, and the HPA axis is the main neuroendocrine system activated by the stress response and therefore cannabinoids [30]. The HPA axis and sympathetic nervous system must balance their roles to mediate the stress. The response to stress is important in survival, but long-term stress can have negative effects on one’s health [31]. Endocannabinoids play their part in allostasis by promoting recovery from stress and further bring back homeostasis of the neurotransmitters, neurohormones, and neuropeptides [32].

Treatment and management strategies

The only treatment identified to fully resolve the symptoms of CHS is cannabis cessation. Numerous studies underscore the effectiveness of this approach, with high success rates reported by Allen et al. [5] and Simonetto et al. [17], where seven out of seven and six out of six patients respectively achieved full symptom resolution upon cessation of cannabis use. In contrast, patients who continued using cannabis experienced persistent symptoms. Furthermore, in cases where patients resumed cannabis use after a period of abstinence, the same symptoms reemerged [5]. This highlights the importance of cannabis cessation in managing CHS, though it is crucial to emphasize that while this intervention resolves symptoms, there is currently no definitive “cure” for CHS; treatment focuses on symptom management.

Phases of Treatment and Hospitalization

Treatment of CHS typically occurs during the hyperemetic phase, which often requires hospitalization due to the severity of symptoms. In this phase, patients frequently experience intractable nausea and vomiting that is unresponsive to conventional antiemetic medications [33]. As noted earlier, hot baths or showers provide temporary relief for many patients. Warm stimulation is hypothesized to transiently activate transient receptor potential vanilloid-1 (TRPV1) in the hypothalamus which would otherwise be downregulated by chronic cannabis use [34]. However, it is essential healthcare providers also address the risk of dehydration and electrolyte imbalances, as these conditions are common in CHS patients and may lead to acute renal failure [27,35]. Fluid resuscitation is therefore a cornerstone of treatment during the hyperemetic phase.

Pharmacological Interventions

In addition to the supportive measures mentioned above, certain pharmacological treatments have shown promise in alleviating symptoms. Haloperidol, a dopamine antagonist and antipsychotic often used off-label as an anti-emetic [36], has demonstrated effectiveness in halting intractable vomiting in CHS patients, with symptom cessation reported as early as one hour after administration [37]. Another promising treatment is topical capsaicin. Capsaicin, applied to the abdomen, has shown success in resolving symptoms in all 15 CHS patients studied in one case report and two case series [38-40]. This effect is thought to be mediated through capsaicin’s interaction with the TRPV-1 receptor, which plays a role in the endocannabinoid system and may modulate nausea and vomiting pathways [41].

Other pharmacologic alternatives were also explored with mixed to unproven efficacy. Corticosteroids, histamine-receptor antagonists, neurokinin-1 receptor antagonists, serotonin receptor antagonists, and opioids have been explored in case reports and preliminary studies but have not yet adequately proven efficacy [42].

Benzodiazepines have been shown to be effective in managing acute symptoms of CHS, particularly for their rapid-acting anti-emetic and anxiolytic properties. These medications exert their effects by inhibiting the medullary and vestibular nuclei in the brain, which play a key role in nausea and vomiting regulation. In emergency settings, benzodiazepines have demonstrated significant efficacy in reducing acute symptoms, providing symptomatic relief when conventional anti-emetics fail. However, their use is approached with caution due to the risk of dependence, especially in patients with a history of substance use. This concern is particularly pronounced in individuals already experiencing sequelae of recreational drug use, necessitating careful patient selection and monitoring when considering benzodiazepines as part of CHS management [43].

Tricyclic antidepressants (TCAs), such as amitriptyline, have shown promising efficacy in the management of CVS and have been explored as a potential prophylactic treatment for CHS. These medications can be initiated during acute episodes of CVS and CHS to help stabilize symptoms and are often continued in a tapered manner following hospital discharge as part of long-term outpatient maintenance therapy. Research suggests that amitriptyline and other TCAs can significantly alleviate CHS symptoms, with remission rates reported in up to 70% of patients. Furthermore, emerging clinical strategies combining gradual cannabis cessation with TCA therapy have demonstrated effectiveness in achieving sustained CHS remission within a period of six to 12 months. This integrative approach underscores the potential role of TCAs in both symptom management and relapse prevention for individuals affected by CHS [44].

Recent developments in cannabinoid research have led to the creation of novel modulators aimed at specifically targeting the CB1 receptors, which are integral to the psychoactive effects of cannabis. When these receptors are activated, they can influence mood and behavior, and in some individuals, may contribute to adverse psychological effects such as depression and suicidal ideation. This raises important concerns about the safety profile of future cannabinoid-based medications and therapies. As research progresses, careful consideration will be needed to balance therapeutic benefits with the potential for harmful side effects, particularly for vulnerable populations. The modulation of CB1 receptors holds promise but also necessitates further investigation to ensure the safety and well-being of patients undergoing such treatments [44-46].

Complications and Caution in Treatment

While several treatments provide symptom relief, it is important to be cautious with certain medications. Narcotic pain medications, for instance, should generally be avoided in CHS patients. Opioids may exacerbate CHS symptoms due to their association with bowel dysfunction, and they could also potentially lead to opioid dependence in chronic users [47]. Additionally, the prolonged use of certain antiemetics, such as ondansetron, may have limited benefit in CHS, further highlighting the need for individualized management plans.

Public health implications

Lack of Public Awareness

While marijuana use is becoming more widespread and socially accepted, there remains a limited public awareness about the adverse effects, including CHS. Many users, particularly younger individuals, may not recognize the potential risks associated with chronic cannabis consumption. Public health campaigns should not only raise awareness about cannabis-related health risks, but also focus on the specific symptoms and potential long-term consequences of cannabis use. Educating consumers, particularly adolescents and young adults, about how to recognize early warning signs of CHS, such as persistent nausea and vomiting or compulsive hot showers, could lead to earlier diagnosis and intervention.

Stigma and Underreporting

A significant public health limitation is the stigma surrounding cannabis use or potential implications with law enforcement, which may discourage individuals from seeking help for cannabis-related health issues. In populations where cannabis consumption is normalized or even celebrated, patients may feel hesitant to disclose their usage or the symptoms they experience, fearing judgment from healthcare providers or the broader community. This barrier to disclosure could result in delayed diagnosis of CHS and other cannabis-related health issues, potentially leading to prolonged suffering or more severe complications. Policies promoting a non-judgmental, open discussion about cannabis use in healthcare settings could help overcome this barrier.

Health System Strain

As cannabis legalization continues to spread across the United States, healthcare systems may face an increasing burden from cannabis-related conditions like CHS. Hospitals and emergency departments, especially in regions with high cannabis use, are likely to see a rise in patients presenting with the characteristic symptoms of CHS. The challenge of diagnosing and managing CHS, coupled with the resource strain of frequent hospital visits, may exacerbate the pressure on healthcare facilities. Adequate training and resources should be provided to healthcare professionals to ensure that CHS is correctly identified and treated, and also to help manage the increased patient load.

Emerging Potency of Cannabis Products

The availability of higher-potency cannabis products, such as concentrates and edibles, poses a growing public health challenge. These products often contain much higher levels of THC than traditional cannabis flower, potentially increasing the risk of developing CHS and other adverse effects. As cannabis products become more potent and accessible, regulatory agencies should consider imposing limits on THC concentration and introducing mandatory labeling that clearly communicates the risks associated with high-potency products.

Regulatory and Policy Gaps

Despite the increasing popularity and legalization of cannabis in many states, there remains a lack of consistent and comprehensive public health policies to address cannabis-related disorders like CHS. Unlike alcohol and tobacco, which have well-established health warnings and regulations, cannabis products are not universally required to include health warnings or educational materials. Public health policies could include mandatory inclusion of warnings on cannabis product labels about the potential for CHS, especially for frequent users, and guidance on safe consumption. Additionally, policies could focus on tracking and reporting cannabis-related health complications to better understand the full scope of CHS and its impact on public health.

Long-Term Societal Costs

Beyond the immediate medical costs associated with CHS treatment, there are long-term societal costs to consider. Chronic cannabis use and its health complications could impact an individual's long-term productivity, education, and career, leading to a broader economic burden on society. As CHS becomes more widely recognized, policymakers should consider its long-term effects on healthcare costs, lost workdays, and the potential need for long-term care or behavioral interventions. These long-term societal costs should be considered when evaluating the broader impact of cannabis legalization.

Research Gaps in Public Health

Public health responses to CHS are hampered by a lack of comprehensive data and research. The rapid expansion of cannabis legalization has outpaced the research into its health impacts, particularly regarding conditions like CHS. There is a clear need for more robust studies to assess the prevalence, demographics, and long-term outcomes of CHS in a variety of populations. This includes not only observational studies but also randomized controlled trials to assess potential treatment options for CHS. Given the rise of CHS cases in regions with legalized cannabis markets, public health agencies should prioritize research funding to fill these gaps and inform future policy.

## Conclusions

CHS is marked by persistent vomiting and ongoing abdominal pain, primarily affecting long-term, daily cannabis users. As cannabis becomes more potent and widely available, CHS is increasingly prevalent. Many patients are hesitant to consider cannabis as the cause of their symptoms, which can delay diagnosis and hinder further research. Moving forward, physicians should be aware of rising cannabis use and identify potential CHS cases to ensure proper treatment and investigation. Further research, particularly at the microscopic level, is essential to better understand this condition.
